# Supplementary material for: Novel Driver Strength Index highlights important cancer genes in TCGA PanCanAtlas patients
Source: PeerJ. 2022 Aug 11;10:e13860. doi: 10.7717/peerj.13860 (PMC9375969; doi:10.7717/peerj.13860)
Supplement: Supplemental Information 2 [file peerj-10-13860-s002.zip › COHORTS/genes plots/detailed/2021_8_16_14_9_distribution_events_detailed_males_6.pdf]

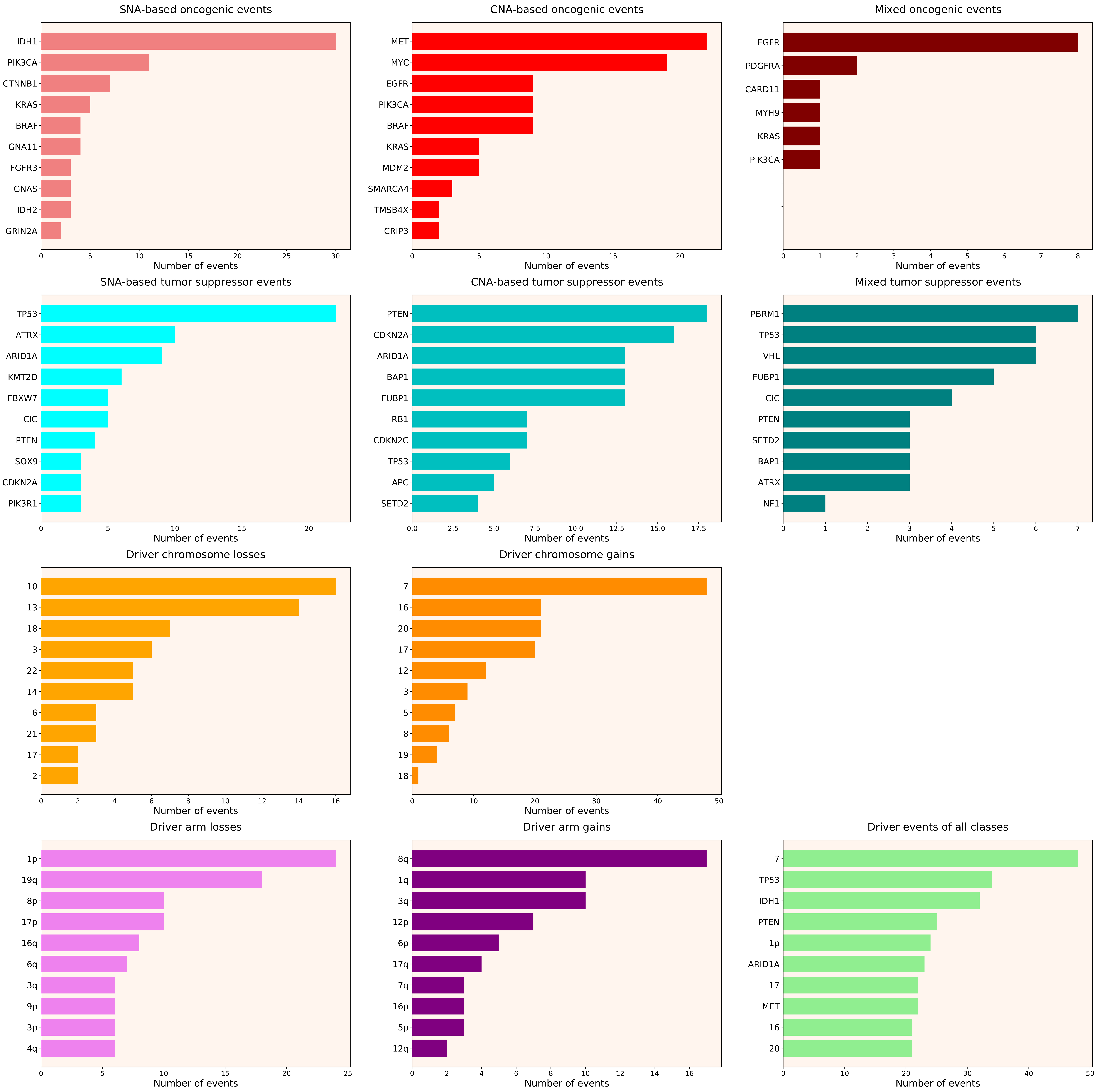

SNA-based oncogenic events
IDH1
PIK3CA
CTNNB1
KRAS
BRAF
GNA11
FGFR3
GNAS
IDH2
GRIN2A
0 5 10 15 20 25 30
Number of events
CNA-based oncogenic events
MET
MYC
EGFR
PIK3CA
BRAF
KRAS
MDM2
SMARCA4
TMSB4X
CRIP3
0 5 10 15 20
Number of events
Mixed oncogenic events
EGFR
PDGFRA
CARD11
MYH9
KRAS
PIK3CA
0 1 2 3 4 5 6 7 8
Number of events
SNA-based tumor suppressor events
TP53
ATRX
ARID1A
KMT2D
FBXW7
CIC
PTEN
SOX9
CDKN2A
PIK3R1
0 5 10 15 20
Number of events
CNA-based tumor suppressor events
PTEN
CDKN2A
ARID1A
BAP1
FUBP1
RB1
CDKN2C
TP53
APC
SETD2
0.0 2.5 5.0 7.5 10.0 12.5 15.0 17.5
Number of events
Mixed tumor suppressor events
PBRM1
TP53
VHL
FUBP1
CIC
PTEN
SETD2
BAP1
ATRX
NF1
0 1 2 3 4 5 6 7
Number of events
Driver chromosome losses
10
13
18
3
22
14
6
21
17
2
0 2 4 6 8 10 12 14 16
Number of events
Driver chromosome gains
7
16
20
17
12
3
5
8
19
18
0 10 20 30 40 50
Number of events
Driver arm losses
1p
19q
8p
17p
16q
6q
3q
9p
3p
4q
0 5 10 15 20 25
Number of events
Driver arm gains
8q
1q
3q
12p
6p
17q
7q
16p
5p
12q
0 2 4 6 8 10 12 14 16
Number of events
Driver events of all classes
7
TP53
IDH1
PTEN
1p
ARID1A
17
MET
16
20
0 10 20 30 40 50
Number of events
